# Brief Motivational Interviewing Delivered by Clinician or Computer to Reduce Sexual Risk Behaviors in Adolescents: Acceptability Study

**DOI:** 10.2196/13220

**Published:** 2019-07-10

**Authors:** Taraneh Shafii, Samantha K Benson, Diane M Morrison

**Affiliations:** 1 Division of Adolescent Medicine Department of Pediatrics University of Washington School of Medicine Seattle, WA United States; 2 Harborview Medical Center University of Washington School of Medicine Seattle, WA United States; 3 School of Social Work University of Washington Seattle, WA United States

**Keywords:** sexual health, risk behaviors, adolescent, healthcare providers, computer-assisted diagnosis, teen health, preventive care

## Abstract

**Background:**

Clinicians are expected to screen their adolescent patients for an increasing number of health behaviors and intervene when they uncover risky behaviors, yet, the clinic time allotted to screen, intervene, and provide resources is insufficient. Brief motivational interviewing (MI) offers succinct behavior change counseling; however, for implementation, clinicians need training, skill, and time. Computerized screening and counseling adjuvants may help clinicians increase their scope of behavioral screening, especially with sensitive topics such as sexual health, and provide risk-reduction interventions without consuming provider time during visits.

**Objective:**

The objectives of this study were to (1) understand the extent to which health care providers use brief MI for sexual health discussions with adolescent patients and (2) assess the acceptability of incorporating a brief MI-based intervention to reduce sexual risk behaviors into their clinical practice delivered by either themselves or a computer.

**Methods:**

At a national medical conference, surveys were administered to clinicians who provide sexual health care to adolescents. They were asked about their current use of MI for sexual risk behavior discussions and their willingness to implement computerized sexual health screening and computerized sexual risk behavior interventions into their clinical practice.

**Results:**

The large majority (87.6%, 170/194) of clinicians already used MI with their patients with less than half (72/148, 48.6%) reporting they had been formally trained in MI. Despite all (195/195, 100.0%) clinicians feeling very or completely comfortable discussing sexual risk behaviors with their patients, the large majority (160/195, 82.1%) reported it would be useful, very useful, or extremely useful for a computerized program to do it all: screen their patients, generate risk profiles, and provide the risk-reduction counseling rather than doing it themselves.

**Conclusions:**

In this study, most clinicians used some form of brief MI or client-centered counseling when discussing sexual risk behaviors with adolescents and are very comfortable doing so. However, the large majority would prefer to implement computerized sexual health screening, risk assessment, and sexual risk behavior interventions into their clinical care of adolescents.

## Introduction

### Background

Clinician sexual health discussions with adolescents remain suboptimal in real-world clinical practice [1-4]. Health care providers continue to search for optimal ways to communicate with adolescents about sexual health and risk behaviors. Some behavioral interventions have been shown to increase the knowledge of risk-reduction strategies (eg, condom and birth control use and negotiating safe sex with partners) and decrease self-reported unprotected sex; however, these interventions were tested in nonclinical settings and with specific populations of adolescents. Such interventions have yet to be tested or implemented in real-world outpatient settings and delivered by clinicians [5-9].

### Barriers to Screening

Even experienced clinicians in busy practices may not have the time to engage adolescents in discussions, which are needed to build rapport and uncover risk behaviors. Adolescents may have concerns about talking face-to-face with clinicians about sex or may not be granted enough time for confidential conversations during their visit [10-12]. Brief motivational interviewing (MI) has gained popularity as a means to engage adolescents in behavior change [13-20]; however, there are barriers to clinicians in adopting MI. It takes time to be trained and become proficient in MI, and effectively using MI requires already precious clinic visit time [21-22].

### Computer-Assisted Screening

Computerized screening with brief MI may serve to alleviate the time burden for health care providers and any discomfort in discussing sensitive health topics for both the clinician and patient. Computer screening improves adolescents’ perceptions of medical visits [23-25]. The literature provides evidence that adolescents may prefer computerized sexual health screening to face-to-face interviews. A study of adolescents seeking care in a pediatric emergency department tested computerized sexual health screening and found that it was acceptable to adolescents, preferable to in-person interviews, and feasible for providers to implement in the emergency department [26]. A personal digital assistant screening tool that screened for several risk behaviors, including unprotected sex, was tested in primary care clinics before adolescent well visits and resulted in higher patient ratings for visit satisfaction, perceived confidentiality, and feeling listened to carefully [23].

### Computerized Interventions

Incorporating sexual behavior risk-reduction interventions into the computerized screening session takes these interventions one step further. Such interventions may be interactive and provide personalized feedback to the adolescent. Only a few computerized sexual health interventions for adolescents have been tested in real-world clinic settings, and these did not assess clinician acceptability of integrating the interventions into clinical practice [27-30]. Existing provider acceptability studies of computerized health screening and interventions are of adult patient populations, have small sample sizes, and may not include sexual health as a risk behavior [31-33]. We are not aware of any large studies assessing clinician willingness to be trained in brief MI for promoting adolescent sexual health. We were likewise unable to identify any studies assessing provider acceptability of incorporating computerized sexual health screening and interventions into visits with their adolescent patients. The objectives of this study were (1) to understand the extent to which health care providers use MI for sexual health with adolescent patients and (2) to assess the acceptability of incorporating a brief MI-based intervention to reduce sexual risk behaviors into their clinical practice delivered by either themselves or a computer.

## Methods

### Recruitment

In March 2009, we administered a 28-item survey to clinicians at a national medical conference, with attendees representing a wide geographic range in the United States. For the purposes of this study, clinicians were asked about sexually transmitted infection (STI) testing and positive STI diagnoses in the past 3 months to characterize their patient population and practice experience. The inclusion criteria were clinicians practicing in the United States who provided sexual and reproductive health care to adolescents. The exclusion criteria were not providing such care to adolescents or being in training. A total of 365 surveys were initially distributed and 18 were omitted from the final denominator (n=347) for the following reasons: the attendee returned the survey blank (n=8); the survey was lost by the participant and a replacement survey was provided (n=8); or the survey was defective because of printing error and was replaced with a corrected survey (n=2). Of the 347 surveys distributed, 81.8% (284/347) were completed. The University of Washington Human Subjects Division approved this study.

### Sample for Analysis

**Figure 1:**
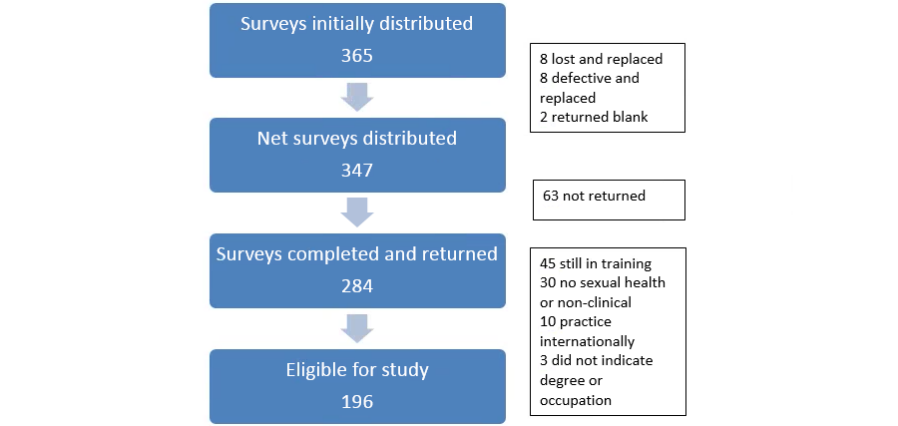
Flow diagram of sample for analysis.

Of the 284 completed surveys, an additional 88 were ineligible because the attendee was in training (n=45), practicing outside the United States (n=10), not providing sexual and reproductive healthcare or not in practice or nonclinical (n=30), or did not indicate their degree or level of training (n=3). A total of 196 clinicians qualified for the study as they provided such care to adolescents, including the diagnosis and management of STIs and unintended pregnancy; identified themselves as medical doctor (MD)/doctor of osteopathy (DO), physician assistant (PA), or nurse practitioner (NP) and were not currently in training (Figure 1). STATA 11.0 by StataCorp LLC, was used for data analysis. *T* tests and chi-square tests were used to evaluate associations.

## Results

### Demographic and Clinical Practice Characteristics

Physicians (MDs and DOs) comprised the majority of the sample (181/196, 92.3%), with (13/196, 6.6%) NPs and (2/196, 1.0%) PAs. In addition, 65.3% (128/196) of them were female. The most common practice type was academic (121/196, 61.7%) with a comparable proportion in private practice (19/196, 9.6%) and public health/community clinics (18/196, 9.2%). Over half (118/195, 60.1%) of the clinicians provided care only to adolescents (defined as ages 11 to 21 years). The number of years providers reported being in clinical practice ranged from less than 1 year to greater than 30 years and was evenly distributed across decades of practice. Through the use of Likert scales, we elicited information about clinical practice and patient population characteristics. The large majority (175/196, 89.3%) of clinicians provided care to 10 or more patients in a typical week. In the past 3 months, the majority (160/195, 82.1%) of clinicians tested 10 or more of their patients for STIs (*Neisseria gonorrhoeae*, *Chlamydia trachomatis*, trichomonas, genital warts, syphilis, and HIV) with the vast majority (178/196, 90.8%) of clinicians reporting 1 or more adolescents testing positive for an STI in the same time period (Table 1).

### Current Use of Motivational Interviewing in Clinical Practice

*All* clinicians reported that they felt at least very comfortable and a large majority felt *completely* comfortable (195/195, 100.0%; missing date, n=1) discussing sexual risk behaviors (eg, inconsistent or lack of condom use/hormonal birth control; multiple partners/concurrency; HIV/STI; unintended pregnancy) with their patients. Although all except 3 clinicians reported feeling that they were at least somewhat effective in changing sexual risk behaviors of their adolescent patients, only 22.1% (43/195) felt very or *completely* effective. Many clinicians reported they saw themselves as more effective at changing patient behaviors than other clinicians.

The vast majority of clinicians were familiar with MI defined in the survey as “… a directive, client-centered counseling style for eliciting behavior change by helping to explore and resolve ambivalence” [34]. The large majority of clinicians (170/194, 87.6%) already used MI with their patients. These 170 clinicians were asked if they were formally trained in MI and of 148 (missing data=22) respondents, less than half, 48.6% (72/148), reported formal training. Clinicians reported having used MI for many health topics, including obesity (93%), smoking (90%), alcohol (82%), substance abuse (87%), and sexual health (96%). Only half (52%) of the clinicians said they used it to discuss injury prevention (bike helmets/seat belts). Most clinicians (140/170, 82.4%) employ MI greater than half the time when discussing sexual health with their patients and feel they are more effective in communicating with their patient when they use MI compared with when they do not (Table 2).

### Motivational Interviewing Acceptability and Feasibility in Practice

The vast majority of clinicians would be willing to use MI or another type of client-centered counseling technique in their practice, if effective at reducing sexual risk behaviors in adolescents. Although 93% of them were willing to attend training for such an intervention, they preferred the length of training be limited to less than 1 day. Clinicians are willing to spend a maximum of 10 min per patient to deliver the intervention. Approximately half the clinicians (103/195, 52.8%) were willing to have follow-up contact with their patients as part of the sexual health intervention. However, 21.0% (41/195) of them would only do so if reimbursed. Clinicians were willing to do at least 1 monthly follow-up with their patients lasting less than 10 min per encounter. Preferred modes of follow-up in order of preference were telephone, email, text message, and social media (Table 3).

**Table 1:** Demographic characteristics of clinicians (N=196).

Demographic characteristic		Statistics, n (%)
**Gender**		
	Female	128 (65.3)
	Male	68 (34.7)
**Clinician type**		
	Medical doctor	181 (92.4)
	Nurse practitioner/physician assistant	15 (7.6)
**Years in clinical practice (N=193), n (%)**		
	≤10	67 (34.7)
	11-20	64 (33.2)
	>20	62 (32.1)
**Patient type (N=195), n (%)**		
	Adolescents only (aged 11-21 years)	118 (60.5)
	Children and adolescents (aged 0-21 years)	30 (15.4)
	All ages (0 through adulthood)	19 (9.7)
	Adolescents and adults (aged 11 years through adult)	28 (14.4)
**Practice type (N=195), n (%)**		
	Academic	121 (62.0)
	Private	20 (10.3)
	Community/public health	23 (11.8)
	Other	31 (15.9)
**Adolescent patients per week**		
	<10	21 (10.7)
	10-29	87 (44.4)
	≥30	88 (44.9)
**STI^a^ tests on patients per 3 months (N=195), n (%)**		
	<10	35 (17.9)
	10-29	59 (30.2)
	≥30	101 (51.9)
**Positive STI^a^ tests per 3 months**		
	<10	123 (62.7)
	10-29	55 (28.1)
	≥30	18 (9.2)

^a^STI: sexually transmitted infection, including chlamydia, gonorrhea, trichomonas, herpes, genital warts, syphilis, and HIV.

**Table 2:** Clinician perspective of sexual risk behaviors and motivational interviewing (MI; N=196).

Clinician perspectives		Statistics, n (%)
**Comfort talking about sexual risk behaviors^a^ (N=195), n (%)**		
	Not comfortable	0 (0)
	Somewhat comfortable	0 (0)
	Comfortable	0 (0)
	Very comfortable	27 (13.8)
	Completely comfortable	168 (86.2)
**Clinician effectiveness in changing sexual risk behaviors (N=194), n (%)**		
	Not effective	8 (4.1)
	Somewhat effective	89 (45.9)
	Effective	68 (35.0)
	Very effective	29 (15.0)
	Completely effective	0 (0)
**Personal effectiveness in changing behavior (N=195), n (%)**		
	Not effective	3 (1.5)
	Somewhat effective	76 (39.0)
	Effective	73 (37.5)
	Very effective	42 (21.5)
	Completely effective	1 (0.5)
**Use of MI with patient**		
	Yes	170 (87.6)
	No	24 (12.4)
**Formally trained in MI (N=170)^a,b^ (n=148), n (%)**		
	Yes	72 (48.6)
	No	76 (51.4)
**Types of behavioral issues addressed**		
	Sexual risk behavior	163 (96.4)
	Obesity	155 (92.8)
	Smoking cigarettes	147 (89.6)
	Drinking alcohol	133 (82.1)
	Substance abuse	140 (87.0)
	Injury prevention (bike helmets/seat belts)	83 (52.2)
**Frequency of use of MI with patients for sexual risk behavior**		
	Never	2 (1.2)
	25% of time	28 (16.5)
	50% of time	41 (24.1)
	75% of time	53 (31.2)
	Almost always	46 (27.0)
**Provider effectiveness in changing behavior when using MI versus when not (N=163), n (%)**		
	Much less effective	1 (0.6)
	Somewhat less effective	4 (2.4)
	No difference	22 (13.5)
	More effective	120 (73.6)
	Much more effective	16 (9.8)

^a^No difference by number practice years or frequency sexually transmitted infection testing.

^b^Remaining survey questions only asked if ever used motivational interviewing, n=170.

**Table 3:** Clinician perspective of self-delivered motivational interviewing (MI).

Clinician perspective		Statistics
**Willing to attend MI training (N=186), n (%)**		
	Yes	174 (93.6)
	No	12 (6.4)
**Maximum length training (N=177), n (%)**		
	≤2 hours	22 (12.4)
	Half day	58 (32.8)
	1 day	54 (30.5)
	≥2 days	43 (24.3)
**Maximum length of MI session with patient (N=192), n (%)**		
	≤5 min	16 (8.3)
	5 min	62 (32.3)
	10 min	67 (34.9)
	15 min	30 (15.6)
	≥20 min	17 (8.9)
**Feasible for clinician follow-up with patient (N=195), n (%)**		
	Yes	103 (52.8)
	No	51 (26.2)
	Only if reimbursed	41 (21.0)
**Maximum length of MI follow-up session (N=152), n (%)**		
	≤5 min	99 (65.1)
	10 min	31 (20.4)
	15 min	12 (7.9)
	≥20 min	10 (6.6)
**Maximum number of monthly follow-up contacts (N=158), n (%)**		
	1	30 (19.0)
	2	39 (24.7)
	3	23 (14.6)
	4-5	29 (18.3)
	6	37 (23.4)
**Follow-up method willing to use, n/N (%)**		
	Phone call	122/149 (81.9)
	Text message	90/144 (62.5)
	Email	124/149 (83.2)
	Social media	30/139 (21.6)

### Computer-Delivered Risk-Reduction Acceptability

The large majority of clinicians (165/192, 85.9%) found it more feasible for a computer to provide the sexual risk behavior screening intervention to their patients rather than themselves and would use a computer-generated sexual risk profile printout to facilitate discussion with their patients. The large majority of clinicians (160/195, 82.1%) also thought it would be useful, very useful, or *extremely* useful for the computer do it all: screen their patient, generate their sexual risk profile, and provide the risk-reduction counseling itself, requiring the provider to review only the findings with their patients afterward.

### Preference for Computerized Risk Screening and Risk-Reduction Counseling

No associations were found when comparing the number of years in clinical practice and comfort discussing sexual risk behaviors with adolescents; being trained in MI; or preferring computerized sexual risk screening and risk-reduction counseling. There was also no association between preference for computerized risk screening and counseling by clinician gender, type of practice, number of patients seen per week, and number of patients tested or testing positive for an STI in the past 3 months (Table 4).

**Table 4:** Clinician perspective of motivational interviewing (MI) and computer-delivered risk reduction for sexual health (N=196).

Clinician perspective		Statistics, n (%)
**If MI sexual behavior risk reduction effectively delivered via clinician would it be feasible for you to do yourself?^a,b^**		
	Yes	183 (95.8)
	No	8 (4.2)
**If sexual behavior risk reduction effectively delivered via computer would that be more feasible for you than doing it yourself?^b,c^**		
	Yes	165 (85.9)
	No	27 (14.1)
**Likeliness to use computer printout of sexual risk behavior profile to facilitate risk-reduction counseling**		
	Not likely	8 (4.1)
	Somewhat likely	34 (17.3)
	Likely	59 (30.1)
	Very likely	68 (34.7)
	Extremely likely	27 (13.8)
**How useful would it be for you if computer generated a printout of sexual risk behavior profile AND provided risk-reduction counseling requiring you to do nothing further OR to simply review the findings with your adolescent patients?^b,d^**		
	Not useful	7 (3.6)
	Somewhat useful	28 (14.4)
	Useful	53 (27.2)
	Very useful	59 (30.3)
	Extremely useful	48 (24.6)

^a^n=191.

^b^No difference by number of years in clinical practice or frequency of sexually transmitted infection testing.

^c^n=192.

^d^n=195.

## Discussion

### Principal Findings

In a survey of clinicians who provide sexual health care to adolescents from varied geographic regions around the United States and a wide range of clinical experience, the vast majority reported being very comfortable discussing sexual health with their adolescent patients. The majority of clinicians reported using MI for sexual health counseling with their patients, although less than half of these reported formally training in MI. Surprisingly, this sample of clinicians espousing such comfort with adolescent sexual health discussions reported that it would be preferable for a computer to do it all: screen for sexual risk behaviors and provide their patients with risk-reduction counseling.

### Comparison With Previous Work

There has been increasing focus on sexual health screening and MI in medical school curricula over the past 2 decades [35-40]. Other studies have found younger clinicians to be more comfortable discussing sexual health and using MI for behavior change as compared with older providers [41,3,1]. However, in this survey, providers with more than 30 years of clinical experience were just as likely as those with less than 10 years of clinical experience to report comfort in talking about sexual health and using MI with patients. The similarity in comfort across respondents with different practice longevity could be because of most clinicians in the study primarily taking care of adolescents and so were comfortable with the population. Also most clinicians were in academic practice, and may be early adopters of evolving clinical practice approaches over the years.

Most providers felt they were at least somewhat effective at influencing the sexual behavior of their patients. This sentiment echoes a qualitative study, with physicians reporting they had influence in the choice of contraception with their female patients [42]. In our study, most respondents considered themselves more effective when using MI than when not, and the majority of them considered themselves more effective at encouraging patients’ behavior change compared with other clinicians. Although there were no studies found in the literature that addressed providers’ perceptions of their effectiveness with MI, there are existing studies that demonstrate clinician use of MI for behavior change to be efficacious in changing health risk behaviors [13-18].

Although respondents were very comfortable discussing sex with adolescents and even felt they were effective at eliciting behavior change, they considered it more feasible for a computer to administer the screening *and* counseling rather than doing so themselves. To our knowledge, this finding is novel in the literature. For this population of providers, preferring a computerized approach to sexual health risk-reduction counseling may reflect time limitations for patient visits rather than reticence to discuss sexual health. Most providers were willing to be trained in an MI sexual risk behavior intervention that includes at least 1 follow-up session; however, 20% of providers indicated they would only do a follow-up session with patients if they were reimbursed, which may also reflect increasing pressures on clinicians for productivity.

### Limitations

A limitation of this study is that we did not define MI in detail or what is required for training and proficiency in true MI. In the survey, we defined MI as “… a directive, client-centered counseling style for eliciting behavior change by helping clients explore and resolve ambivalence” [34]. It is possible that participants have different definitions for and experience in the use of MI, which may have biased the responses to questions about the use of and training in MI. In addition, the proposed computerized screening and intervention was theoretical, so clinicians were not providing feedback on a tangible product for which they may have different opinions. As most clinicians practiced in academic settings, the findings may not be generalizable to clinicians in other practice types. The decision was made to focus on clinicians practicing in the United States to account for the large variation worldwide in attitudes toward adolescent sexual and reproductive health and clinical practice. The authors acknowledge that this was a missed opportunity to learn about international clinician practice.

MI has gained increasing popularity over the past decade since this study. *Brief* MI is used for many different health behaviors, and we anticipate an even higher acceptability by medical providers. However, the issue of lack of provider time with patients has also escalated over the past decade. These data are relevant as providers have not yet found an answer and continue to strategize on how they can provide comprehensive health care in the limited minutes they have for adolescent patient visits. Such an intervention as presented in this study is a possible solution.

### Conclusions

Clinicians are increasingly pressed for time when providing care to patients and researchers and practitioners have not yet found the most effective way to consistently discuss sexual health with adolescents or promote healthy sexual behaviors. Computerized interventions, which incorporate both behavioral screening and risk-reduction counseling, may provide solutions to both issues. The development of computerized health interventions is a rapidly growing field and further research is needed to create and test such interventions in real-world clinical practice.

## Abbreviations

DO: doctor of osteopathy
MD: medical doctor
MI: motivational interviewing
NP: nurse practitioner
PA: physician assistant
STI: sexually transmitted infection
